# How confident are pharmacists in providing pharmaceutical care on anticoagulants? A cross-sectional, self-administered questionnaire study in Borneo, Malaysia

**DOI:** 10.1186/s40545-021-00377-w

**Published:** 2021-11-09

**Authors:** Sze Ling Tan, Zai Yang Yong, Jerry Ee Siung Liew, Hadzliana Zainal, Sania Siddiqui

**Affiliations:** 1 Pharmacy Department, Hospital Queen Elizabeth II, Kota Kinabalu, Sabah Malaysia; 2 Pharmacy Department, Sarawak General Hospital, Kuching, Sarawak Malaysia; 3 Pharmacy Department, Queen Elizabeth Hospital, Kota Kinabalu, Sabah Malaysia; 4 School of Pharmaceutical Sciences, Universiti Sains Malaysia, Penang, Malaysia

**Keywords:** Anticoagulants, Pharmaceutical care, Confidence, Borneo Malaysia

## Abstract

**Background:**

Anticoagulants are the cornerstone therapy for the management of venous thromboembolism (VTE) and atrial fibrillation (AF). Pharmacists should be confident and equipped with the skill and updated knowledge in managing anticoagulation therapy.

**Objective:**

To explore self-reported confidence level of pharmacists, perceived reasons influencing their confidence and socio-demographic associated with high confidence level in the area of anticoagulation.

**Methods:**

A cross-sectional, self-administered questionnaire survey was carried out among fully registered pharmacists who work in selected government hospitals and clinics in Borneo, Malaysia, from January 2019 to February 2020.

**Results:**

Overall, responses from 542 fully registered pharmacists were obtained. Proportion of respondents who claimed confident in providing necessary information to patient receiving warfarin (*n* = 479, 88.3%) was significantly higher (*p* < 0.001) compared to low molecular weight heparins (*n* = 317, 58.5%) and direct oral anticoagulants (*n* = 211, 38.9%). Respondents’ perceived reasons that may influence their confidence level include experience in dealing with anticoagulants’ cases (*n* = 469, 86.5%), knowledge on anticoagulants (*n* = 394, 72.7%) and knowledge on diseases needing anticoagulation therapy (*n* = 311, 57.4%). Practising as ward pharmacist and “always” dealing with anticoagulants during their practice were the socio-demographic that significantly associated with high confidence level of pharmacist in providing pharmaceutical care on all types of anticoagulants (*p* < 0.05).

**Conclusion:**

Pharmacists were found more confident in providing pharmaceutical care on warfarin compared to low molecular weight heparins and direct oral anticoagulants. Continuous educational and training programmes on the use of anticoagulants should be carried out to enhance pharmacists’ confidence in supporting patients’ care.

**Supplementary Information:**

The online version contains supplementary material available at 10.1186/s40545-021-00377-w.

## Introduction

Anticoagulants are the cornerstone therapy for the management of venous thromboembolism (VTE) and atrial fibrillation (AF) [1]. VTE is one of the leading cause of mortality worldwide and estimated to be the third most common vascular disease after myocardial infarction and stroke [2]. The actual incidence of VTE in Malaysia remains unknown, however literatures showed an increase trend in the incidence rates in both Western and Asian countries [3, 4]. On the other hand, AF is the commonest sustained cardiac arrhythmia that affects 1% of the global population, and approximately 0.54% in Malaysia population [5]. It is known that AF increases the risk of stroke and based on CHA_2_DS_2_-VASc (congestive heart failure, hypertension, age ≥ 75 years, diabetes mellitus, stroke or transient ischaemic attack, vascular disease, age 65 to 74 years, sex category) score, patient should receive oral anticoagulant if one does not fall into the “low risk” category [6]. Various guidelines have recommended the use of anticoagulants such as vitamin K antagonists (VKAs) or direct oral anticoagulants (DOACs), in some circumstances, low molecular weight heparins (LMWHs), as part of the pharmacotherapy in management of AF and VTE used to reduce thromboembolism complications, risk of stroke and mortality [7–13]. Warfarin is the oldest and most widely used vitamin K antagonist [14]. Warfarin has a narrow therapeutic index which requires frequent International Normalised Ratio (INR) monitoring and known to have multiple interactions with drugs, food and supplements [15]. The emergence of DOACs since last decade has gained popularity as these newer agents have demonstrated similar efficacy but a better safety profile compared to warfarin in management of AF [16–20] and VTE [21, 22]. Due to the advantages of DOACs such as minimal anticoagulation monitoring needed and less documented drugs/supplements-related interactions, the usage of DOACs has steadily increased. The Malaysian Statistics on Medicines (MSOM) has shown that the usage of DOACs such as dabigatran and rivaroxaban is increasing steadily from year 2011 to 2014 in both private and government sector [23]. In Malaysia’s government hospitals and clinics, pharmacist-initiated medication counselling session are given to patients who are newly started on warfarin, LMWHs (enoxaparin) and DOACs (dabigatran, rivaroxaban, apixaban) [24, 25]. Literatures have proven that the involvement of pharmacists in anticoagulation management has positive impact in improving safety and efficacy outcomes in patients taking warfarin [26] or receiving DOACs [27]. To date, only two published questionnaire-based studies have assessed the confidence level among pharmacists in dealing with anticoagulants. The first multinational survey was conducted by Papastergiou et al. [28], involving 4212 pharmacists from 18 countries (not including Malaysia). This study showed that pharmacists’ confidence level was significantly lower (*p* < 0.001) when advising patients on DOACs compared to VKA. Another study was carried out by Hamedi et al. [29] aimed to assess the community pharmacists’ current practice, knowledge and confidence in supporting patient’s adherence on oral anticoagulants for stroke prevention in atrial fibrillation in London via online survey where 257 community pharmacists responded. Results documented that pharmacists were less confident in their knowledge, skills and access to resources for DOACs than for VKA (*p* < 0.005). The unfamiliarity and minimal exposure to DOAC is reflected in their knowledge and confidence. Both studies did not assess the confidence level on advising LMWHs even though LMWHs are commonly used as parenteral anticoagulant especially in in-patient care.

Up to the present, warfarin, LMWHs such as enoxaparin, and DOACs such as dabigatran, rivaroxaban, apixaban are approved by Ministry of Health medicines formulary (MOHMF) Malaysia [30] to be used as part of the management in AF and VTE. Pharmacists, whether involved directly or indirectly with patient care, should be confident and well equipped with the skills and updated knowledge in managing anticoagulation therapy. However, there is lack of assessment on the confidence level of pharmacists in providing pharmaceutical care on anticoagulants to these patients. Thus, this study aimed to assess the self-reported confidence level of pharmacists in Borneo, Malaysia, in terms of providing necessary information on 3 types of anticoagulants (warfarin, LMWHs and DOACs) and discussing various aspects of anticoagulation therapy with patients. This study also described the socio-demographic associated with high confidence level, respondents’ perceived reasons that influencing their confidence as well as education needs in the area of anticoagulation therapy in order to identify the potential methods to fill in the gap.

## Methods

### Study methods

This was a cross-sectional, self-administered questionnaire study which was carried out in Pharmacy Department of government hospitals and clinics in Borneo (Sabah and Sarawak) from January 2019 until February 2020. Sample size of 526 respondents is required with expected 53% of pharmacist claim confident in providing pharmaceutical care on DOACs [28], also with precision of 2.5% and 95% confidence interval (refer Additional file 1: Appendix for details) [31]. In order to ensure there was adequate representation from both hospitals and clinics in Borneo, all facilities in Sabah and Sarawak were being stratified into six strata, respectively: hospital (main zone), clinic (main zone), hospital (district 1), clinic (district 1), hospital (district 2), clinic (district 2). Then, simple random sampling was employed to select facilities in each strata to meet the responses needed. All the fully registered pharmacists (FRPs) working in the selected facilities were approached via either email or by investigators; upon consent, they were required to fill up the questionnaire form and return to investigators. This study was approved by the Medical Research and Ethics Committee, Malaysia, with the identification code of NMRR-18-2821-44278 (IIR) for Sabah and NMRR-19-3270-51648 for Sarawak.

### Instrument

The questionnaire used in this study comprises of five domains: (i) socio-demographic; (ii) self-report confidence level in providing necessary information on 3 types of anticoagulants (warfarin, LMWHs and DOACs) and discussing various aspects in anticoagulation therapy using 4-point Likert scale ranging from not confident at all to very confident, (iii) perceived reason(s) influencing confidence level; (iv) ways used by pharmacists to obtain latest information on anticoagulants; (v) education needed in the area of anticoagulation therapy. Domain (ii) and (v) of the questionnaire were adapted from the validated questionnaire (refer Additional file 1: Appendix for psychometric properties) used in multinational survey by Papastergiou et al. [28] after obtaining authors’ consent. Content validation using Delphi technique and pre-test were carried out among university lecturers and hospital pharmacists to ascertain the comprehensive and user-friendliness of questionnaire.

### Data analysis

Analysis was conducted using STATA/SE 12.0 (StataCorp, College Station, TX, USA). Continuous variables were presented in mean (standard deviation, SD) or median (interquartile range, IQR) depending on the normality of data distribution. Categorical data were presented as frequency and percentage. Before conducting univariate analysis, data were explored to check for any errors and incorrect data entry. Categorical variables were merged if there were cells with zero values. Proportion of confidence across 3 types of anticoagulants were analysed using Chi-square, then Bonferroni correction was done for multiple comparisons. For this, a *p* < 0.017 (3 comparisons) was considered as statistically significant. Subsequently, logistic regression analysis was adopted.

We aimed to determine the factors associated with pharmacist's confidence level (confident vs not confident) in advising anticoagulants (warfarin). The first step in fitting the model was to conduct univariable logistic regression. The relationship between each socio-demographic and dependent variable (pharmacist’s confidence level in advising warfarin) was evaluated. Variables that were found to be statistically significant (*p* < 0.25) and additional variables that might plausibly affect the dependent variable were considered as candidate variables in multiple logistic regression. In order to identify the factors that would provide the most parsimonious model within the constraint of current collected data, best subsets selection using Furnival–Wilson leaps-and-bounds algorithm [32] was employed. This algorithm organised all subsets of factors in tree structures and evaluated them by skipping (or “leaping”) over a subset that was not optimal. The subsets of factors created were all possible combinations of socio-demographic variables (age, working experience as pharmacist (months), place of practice, zone of practice, department of practice, warfarin MTAC involvement and frequency of warfarin used in practice). The best subset was determined based on smallest Bayesian information criterion (BIC) and Akaike’s (1974) information criterion (AIC) value as well as the largest log likelihood. After executing the algorithm, a total of 7 subsets of factors were generated. The subset that consisted of department of practice (*X*_1_) and frequency of warfarin used in practice (*X*_2_) were found to have the smallest BIC (364.3553) and AIC value (351.4751). This subset provided the best possible explanation of the dependent variable (pharmacist’s confidence level in advising warfarin). Hence, a preliminary main effect model was obtained.

Subsequently, the adequacy of the preliminary main effect model was studied. This included checking for interaction effect, multicollinearity and analysis of residuals. The presence of an interaction effect was assessed by forming a new variable that was the product of *X*_1_ × *X*_2_ and including this product term in the regression, along with the original predictor variables. No interaction effect was detected as the product of *X*_1_ × *X*_2_ was statistically not significant (*p* > 0.05). For multicollinearity assessment, variance inflation factor (VIF) for each factor was calculated and VIF of more than 5 was deemed to represent critical level of multicollinearity. Moreover, high correlation among predictors may also be presented if multicollinearity was an issue. A large inflated standard error, usually more than 5 would be observed. From our analysis, VIF for both factors were less than 5 (VIF = 1, respectively, for each variable) and their standard errors were small (department of practice, OR 5.56, SE = 3.39; frequency of warfarin used in practice, OR 4.71, SE = 1.43). Therefore, no multicollinearity was detected. As for the analysis of residuals, the main aim was to detect potential outliers, especially influential outliers. The outliers are deemed influential if extreme values can unduly influence the results of the analysis and lead to serious ramifications on the validity of the inferences. Potential outliers were detected from standardised residuals. Cook’s distance was calculated to determine whether the outliers were influential enough. An observation was considered as an outlier if it had a standardised residuals of larger than 2 or smaller than -2 and the outliers were influential if Cook’s distance was greater than 1. Residual analysis showed that an observation (Case ID: 5) fulfilled criteria of being an influential outlier. Hence sensitivity analysis was conducted. A model that included this observation was compared with a model without this observation and it was noted that a significant improvement in parameter estimates and reduction in standard error were notable. Thus, this observation was removed from the final model (*n* = 541).

For model fit assessment, Pearson *χ*^2^ goodness-of-fit (GOF) test and Hosmer–Lemeshow test were carried out. Test results obtained showed a large *p*-value of more than 0.05 for both tests. This indicated that the data fit the logistic regression model well.

Finally, the performance of the model was evaluated by examining the ability to classify and discriminate between the two levels of response variable. The classification tools utilised were classification table and area under the receiver operating curve (ROC curve). A cut-off value of >70% indicates excellent discriminating power between the two levels of the response variable. Analysis result showed that the overall correctly classified percentage and area under the ROC curve were 88.54% and 71.09%, respectively. Hence, the final model was obtained. The parameter estimates from the model were interpreted as odds ratio (OR).

Similar ways in conducting logistic regression were repeated to determine socio-demographics that may be associated with pharmacist’s confidence level in advising LMWHs and DOACs. All assumptions of logistic regression (adequacy of the logistic regression model, model fit assessment and model discrimination ability) were checked. Logistic regression assumption test results were presented concomitantly with each model plot (Fig. 2). All probability values were two-sided. A *p*-value of less than 0.05 was considered statistically significant.

## Results

### Characteristics of respondents

A total of 542 FRPs (260 from Sabah and 282 from Sarawak) responded to the questionnaire. Table 1 summarises the characteristics of all respondents. Among 542 respondents, majority were female (*n* = 428, 79.0%) with median age of 30 years old and median month of working as pharmacist of 61.5 months. Warfarin remains the most common anticoagulant encountered by pharmacists during their practice with 58.3% (*n* = 316) respondents claimed they “always” deal with it during their practice.

**Table 1:** Socio-demographics of respondents

Characteristics	*n* (%)
Gender	
Female	428 (79.0)
Male	114 (21.0)
Age (year)	30 (4)^a^
Working experience as pharmacist (month)	61.5 (62)^a^
Zone of practice	
Main town	251 (46.3)
District 1	180 (33.2)
District 2	111 (20.5)
Place of practice	
Hospital	395 (72.9)
Clinic	147 (27.1)
Place of practice according to zone	
Hospital (Main Zone)	189 (34.9)
Hospital (District 1)	126 (23.2)
Hospital (District 2)	80 (14.8)
Clinic (Main Zone)	62 (11.4)
Clinic (District 1)	54 (10.0)
Clinic (District 2)	31 (5.7)
Department of practice	
Outpatient	269 (49.6)
Ward	95 (17.5)
Inpatient	74 (13.7)
Administrative	11 ( 2.0)
Others	93 (17.2)
Warfarin MTAC involvement	
Never	395 (72.9)
Previously	76 (14.0)
Currently	71 (13.1)
Anticoagulant deal by respondent in practice	
Warfarin	
Never	25 (4.6)
Rarely	58 (10.7)
Sometimes	143 (26.4)
Always	316 (58.3)
LMWHs	
Never	62 (11.4)
Rarely	167 (30.8)
Sometimes	192 (35.4)
Always	121 (22.3)
DOACs	
Never	84 (15.5)
Rarely	224 (41.3)
Sometimes	184 (33.9)
Always	50 ( 9.2)

*DOACs* direct oral anticoagulants, *LMWHs* low molecular weight heparins, *MTAC* medication therapy adherence clinic

^a^Data skewed to the right, *p* < 0.05 in Kolmogorov–Smirnov statistic with a Lilliefors significant level

### Confidence level of respondents

**Fig. 1:**
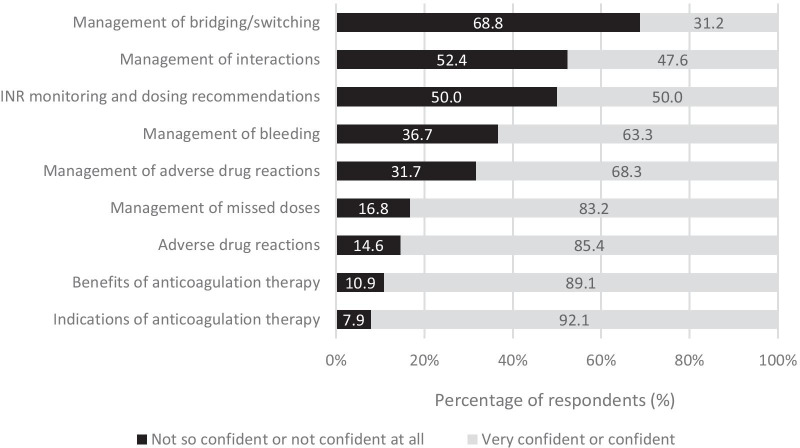
Distribution of responses (*n* = 542) according to their confidence level in different aspects of anticoagulation therapy

The distribution of respondents’ confidence level according to the type of anticoagulants is shown in Table 2. It was found that there were significant differences in the proportion of confidence across the 3 types of anticoagulants (*Χ*^2^ = 285.1; *df* = 2, *p* < 0.001). Bonferroni post hoc tests has shown that the proportion of respondent who reported confident in providing necessary information to patient receiving warfarin was significantly higher than LMWHs (*Χ*^2^ = 124.12; *df* = 1; *p* < 0.001) and DOACs (*Χ*^2^ = 286.42; *df* = 1; *p* < 0.001). Similarly, respondents reported higher confidence when advising patients on LMWHs than DOACs (*Χ*^2^ = 41.49; *df* = 1; *p* < 0.001). The distribution of respondents’ confidence according to the various aspects of anticoagulation therapy is demonstrated in Fig. 1. It was clearly shown that respondents generally felt confident when discussing about indications (*n* = 499, 92.1%) and benefits of anticoagulation therapy (*n* = 483, 89.1%) with patients. However, at least half of the respondents were not confident when it comes to INR monitoring and dosage adjustment (*n* = 271, 50.0%), management of interactions (*n* = 258, 47.6%) and management of bridging/switching from one anticoagulant to another (*n* = 169, 31.2%).

**Table 2:** Distribution of pharmacists’ confidence level according to type of anticoagulants

Type of anticoagulant	Confident *n* (%)	Not confident *n* (%)	_*Χ*_^2^	*p* value*
Warfarin	479 (88.3)	63 (11.7)	285.1^a^	< 0.001
LMWHs	317 (58.5)	225 (41.5)		
DOACs	211 (38.9)	331 (61.1)		

*DOACs* direct oral anticoagulants, *LMWHs* low molecular weight heparins

^a^Chi-square test for independence; 0 cells (0%) have expected count less than 5

^a^The minimum expected count is 206.33

When respondents were asked on their perceived reason(s) which influencing their confidence level, most of the respondents reported that their experience in dealing with anticoagulation cases (*n* = 469, 86.5%), knowledge on anticoagulants (*n* = 394, 72.7%) and knowledge on the diseases requiring anticoagulation therapy (*n* = 311, 57.4%) were the main reasons influencing their confidence level in providing pharmaceutical care to patients regarding anticoagulants. About 10% (*n* = 59) of respondents claimed that the previous unfavourable experiences with anticoagulants affect their confidence level. When asked about the source of information used by respondents to obtain latest information regarding anticoagulants, more than half of the respondents utilised updated local/international guidelines (*n* = 366, 67.5%), peer discussion (*n* = 333, 61.4%) and medical websites (*n* = 283, 52.2%). Non-medical website(s) (*n* = 53, 6.5%) was the least preferred choice of respondents for the obtainment of information. Up to 93.5% (*n* = 507) respondents would like to receive additional education in the area of anticoagulation, particularly on the bridging/switching (*n* = 460, 90.7%), management of bleeding (*n* = 393, 77.5%) and drug–drug, drug–food interaction (*n* = 390, 76.9%). Besides that, few respondents commented that they were interested in the drug choice and dosing in special population.

### Socio-demographics associated with confidence level of respondents in providing necessary information to patient receiving warfarin, LMWHs and DOACs

**Fig. 2:**
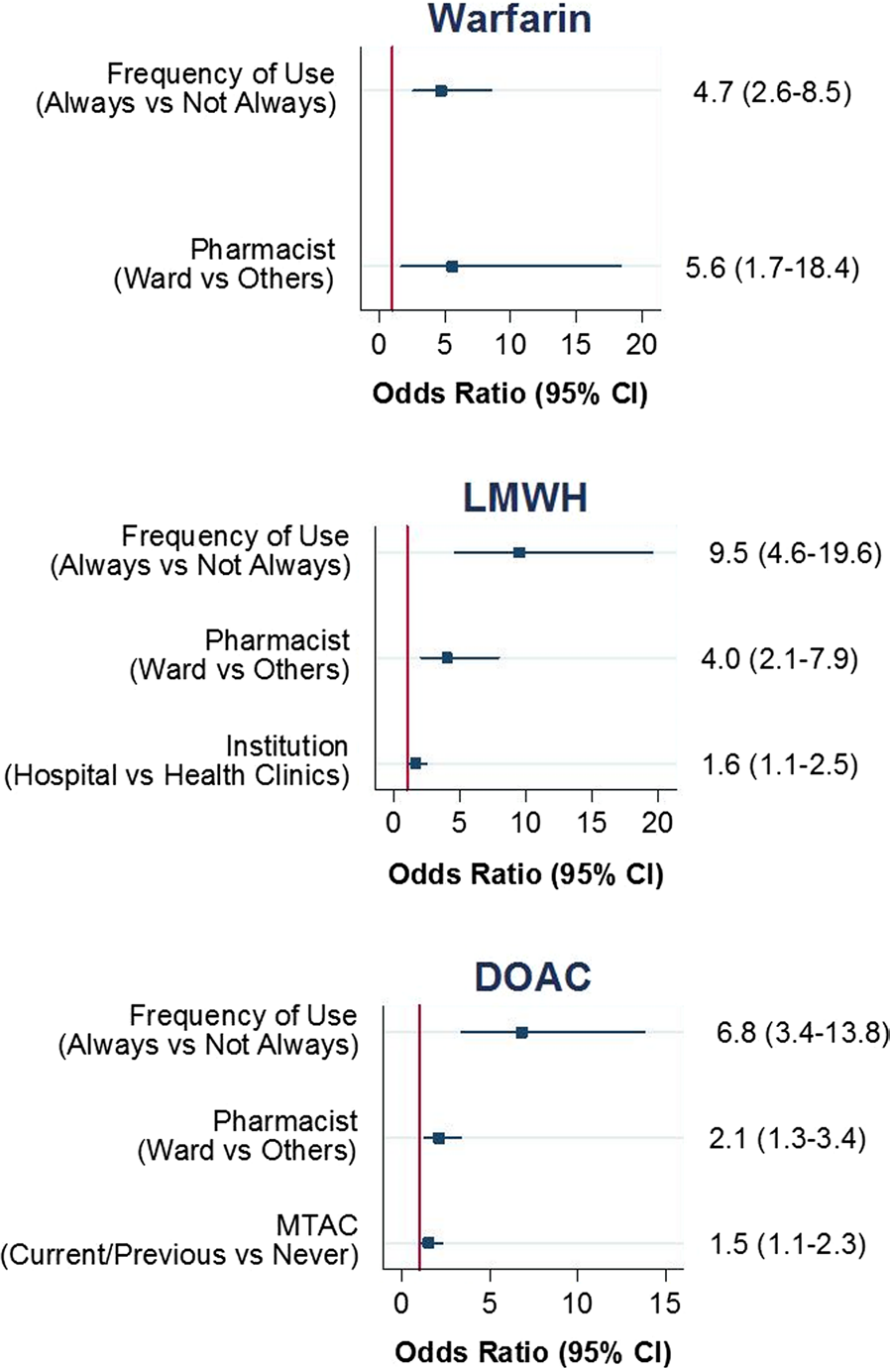
Socio-demographics that associated with pharmacists’ confidence in warfarin, LMWHs, DOACs; no multicollinearity and interaction detected. Hosmer–Lemeshow test, (*p* > 0.05 for all), classification table (overall correctly classified percentage = 88.54%, 66.2%, 67.16%), area under the ROC curve (71.09%, 73.4%, 64.63%), AIC value (351.5, 623.6, 682.03), BIC value (364.36, 640.8, 699.21) and pseudo *R*^2^ (0.1032, 0.1632, 0.069) were applied to check the model fit

Figure 2 reports the socio-demographics that are associated with high confidence level in providing necessary information to patient receiving warfarin, LMWHs and DOACs using multiple logistic regression. Frequent user and ward pharmacists have been consistently found to be more confident in providing care on all anticoagulants compared to non-frequent user and non-ward pharmacists when adjusted for all variables. Apart from this, hospital pharmacists, compared to respondents who are working in clinics, has 1.6 times of odds (95% CI 1.1; 2.5, *p* < 0.05) of being more confident in providing care on LMWHs. Those who had warfarin medication therapy adherence clinic (MTAC) exposure has 1.5 times of odds (95% Cl 1.1; 2.3) of greater confidence in dealing with DOACs compared to those who had never involved in warfarin MTAC previously.

## Discussion

To the best of our knowledge, this is the first questionnaire survey ever conducted in Malaysia that assessed the confidence of pharmacists in dealing with anticoagulants. Overall, pharmacists working in Borneo have displayed a higher confidence level in providing necessary information to patient receiving warfarin compared to LMWHs and DOACs (88.3% vs 58.5% vs 38.9%, respectively). The findings were similar to a multinational survey conducted among pharmacist working in 18 countries [28], whereby the mean proportion of confidence with vitamin K antagonist and DOACs were reported as 78.2% (ranging from 54.5 to 97.0% among studied countries) and 53% (ranging from 21.6 to 82.3% among the studied countries), respectively. In addition, Hamedi et al. [29] also reported that approximately 40% (*n* = 102) of the community pharmacists in London felt uncertain or lacked of confidence in their knowledge on DOAC, compared to vitamin K antagonist (*n* = 59, 23%). The higher confidence level reported by respondents in dealing with warfarin was not surprised as warfarin has been available for decades and is still widely use in the government healthcare facilities. Moreover, warfarin counseling is a part of module for pharmacist during pre-registration training that possibly lend them in attaining higher confidence in providing pharmaceutical care to patients receiving warfarin compared to other anticoagulants.

Although DOACs demonstrated similar efficacy and a better safety profile compared to warfarin, medication adherence towards DOACs is very important as DOACs has relatively short half-lives and lack of routine therapeutic monitoring [25]. As pharmacists known as the most accessible health care providers, can play a role in management and monitoring of DOACs therapy [25]. However, the low confidence level in dealing with DOACs reported in this study is alarming as the usage of DOACs would be expected to increase in accordance to the latest guidelines’ recommendation [7, 8] where DOACs are strongly recommended over warfarin in DOAC-eligible AF patients. Apart from that, Pharmaceutical Services Program, Ministry of Health Malaysia has recently published the Anticoagulation Medication Therapy Adherence Clinic (AC-MTAC) Protocol 2020 [24] and with the ongoing transition from Warfarin Medication Therapy Adherence Clinic (WMTAC) to AC-MTAC, pharmacists will be expected to provide care to more DOACs patient in the future. Since more than half of the respondents agreed that knowledge about anticoagulants and knowledge on diseases requiring anticoagulation therapy were the factors affecting their confidence level, pharmacists should be proactive in enhancing their knowledge and skill towards management of anticoagulation (especially DOACs).

With regards to the confidence level of respondents in discussing various aspects of anticoagulation therapy with patients receiving anticoagulants, our results were consistent with the findings reported by Papastergious et al. [28], where more than half of the respondents claimed not so confident or not confident at all in management of bridging/switching from one anticoagulant to another, management of interactions as well as INR monitoring and dosing recommendations. Pharmacists generally doubt themselves in these areas as these are more complicated and require good clinical knowledge. Thus, these areas should be focused in future anticoagulation training workshop or education sessions. Local/international guidelines, peer discussion and reference to medical website(s) were the preferred way for pharmacists to obtain latest information on anticoagulation therapy. These results provided a general background on the preferred source of information by pharmacists and may aid in future planning in updating information about anticoagulants.

More than 95% of pharmacists express their interest in receiving additional education on different aspects in the area of anticoagulation therapy. Majority (over 90%) were interested in receiving education on bridging/switching of anticoagulants, followed by 77.5% pharmacists who want to receive education in the management of bleeding associated with anticoagulants. The findings were in line with the published study where 68.2% of pharmacist wanted to receive education on bleeding management, whereas 66.0% were reportedly interested in getting additional education on management of switching [28]. This finding reflected the perceived need of education by pharmacist which they found difficult while advising patients on anticoagulants and could be used in developing relevant education programmes to fill gaps in their knowledge and boost their confidence.

With regard to the evaluation of the socio-demographics associated with the pharmacists’ confidence in providing pharmaceutical care on anticoagulation therapy, it was found that ward pharmacists and pharmacists who always encountered anticoagulants in their practice were consistently more confident in providing anticoagulant-related pharmaceutical care. Ward pharmacists often deal with a variety of anticoagulation cases and involve in active communication with patients and their families; this leads them in acquiring better confidence level when providing care to patients. As for pharmacists who always deal with anticoagulants, they are likely to have more experience in dealing with the anticoagulants cases and this is being proven where majority of pharmacists (*n* = 223, 85.8%) agreed that the experience in dealing with anticoagulant cases was the pivotal factor affecting their confidence level. Given the complexity of therapies and complications associated with VTE and AF, regardless of the type of anticoagulant used, the exposures to different case scenarios and regular training workshop or education sessions maybe required to enhance pharmacist’s knowledge and confident. Pharmacist’s prior or current medication therapy adherence clinic (MTAC) exposure contributed to a higher confidence level when dealing with DOACs. In Malaysia, pharmacists are required to undergo a 2-week warfarin MTAC training and pass examination before being recruited in warfarin MTAC services [33]. This sets a good foundation for pharmacists and with routine MTAC activities such as patient review and counselling, these are not only helpful in increasing patient adherence towards anticoagulants but may also substantially increase pharmacists’ own knowledge and ultimately their confidence in the area of anticoagulation therapy [24, 34].

### Strength and limitation

Strength of this study was the robust sampling that was able to have sufficient representation from all levels of practising pharmacists such as urban and suburban, hospital and clinics as well as various levels of pharmacist position in government setting. These enable us to generalise the findings among government pharmacists working in Borneo (both Sabah and Sarawak). Even though the findings may not be representative of the confidence level of pharmacist in the area of anticoagulation working in the other states of Malaysia, this study could provide a basis for conducting a large-scale national survey for the generalisation of the results. This study was cross-sectional in nature, thus unable to identify changes in the confidence level of pharmacists over time in the long run. As responses were reported from pharmacist working in the government settings only, confidence level of pharmacist working in private sectors, retail or in community pharmacies remains unknown and should be evaluated by future studies.

## Conclusion

This study assessed the confidence level in providing pharmaceutical care on commonly used anticoagulants, namely warfarin, LMWHs and DOACs in Borneo, Malaysia. Results of this study suggested that Borneo pharmacists has higher confidence level in providing pharmaceutical care to patients receiving warfarin compared to low molecular weight heparins and direct oral anticoagulants. Keeping abreast with current guidelines through structured and continuous educational programme is urgently needed to ensure the safety and efficacy of anticoagulation therapy.

## Supplementary Information

**Additional file 1.** Supplementary appendix for sample size calculation, questionnaire and its' original psychometric properties.

## Data Availability

The datasets used and/or analysed during the current study are available from the corresponding author on reasonable request.
