# Supplementary material for: How confident are pharmacists in providing pharmaceutical care on anticoagulants? A cross-sectional, self-administered questionnaire study in Borneo, Malaysia
Source: J Pharm Policy Pract. 2021 Nov 9;14:97. doi: 10.1186/s40545-021-00377-w (PMC8576986; doi:10.1186/s40545-021-00377-w)
Supplement: Supplementary file 1 — Additional file 1. Supplementary appendix for sample size calculation, questionnaire and its' original psychometric properties. [file 40545_2021_377_MOESM1_ESM.docx]

**Supplementary Appendix**

**Sample size calculation**

According to study done by Papastergiou et al.^1^, the mean prevalence of pharmacists very confident and confident in providing pharmaceutical care on VKAs and DOACs were 78.2% and 53% respectively. Thus, expected proportion (P) would be either 0.78 or 0.53. Based on the pharmaceutical services in Sabah and Sarawak, total number of pharmacists (N) working in public hospitals and government health clinic in Borneo was estimated to be 800. In this study, a 95% level of confidence and precision (d) of 0.025 was used.

Using “sample size calculator for prevalence studies” with finite population correction^2^ (formula as below), the calculated sample size was 456 and 526 for expected P of 0.78 and 0.53 respectively. A higher sample size is chosen in order to meet the objective thus at least 526 respondents are required in this study.


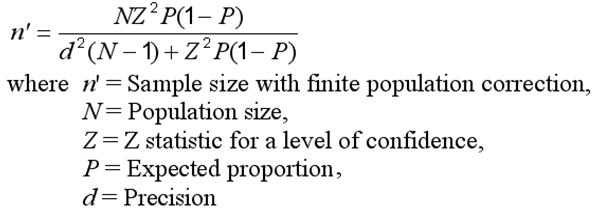


References:

1. Papastergiou J, Kheir N, Ladova K, Rydant S. Pharmacists ’ confidence when providing pharmaceutical care on anticoagulants , a multinational survey. Int J Clin Pharm 2017; doi: 10.1007/s11096-017-0551-2.

2. Naing L, Winn T, Rusli BN. Practical Issues in Calculating the Sample Size for Prevalence Studies. Archives of Orofacial Sciences 2006; 1: 9-14.

QUESTIONNAIRE

| **Participant ID: _________________** |
| --- |

**Section A: Socio-demographic**

Please tick (√) ONE box only or write in the box where appropriate.

1. Gender : ( ) Male
    : ( ) Female
2. Age : _______ years old
3. Years of experience as pharmacist : ________ year(s) _______ month(s)
4. Place of practice currently : ( ) Hospital
    : ( ) Government health clinic
5. Department of practice currently : _________________
6. Are you involved in MTAC Warfarin?: ( ) Yes currently
    : ( ) Not now but yes previously
    : ( ) Never
7. How frequent do you encounter anticoagulants as below in your practice?

|  | Never | Rarely | Sometimes | Always |
| --- | --- | --- | --- | --- |
| Warfarin |  |  |  |  |
| Low molecular weight heparins |  |  |  |  |
| Direct oral anticoagulants |  |  |  |  |

**Section B: Confidence level in providing pharmaceutical care to patients receiving anticoagulants**

Please choose the answer that most accurately reflects your confidence level in order to ensure that patients are treated appropriately with anticoagulants. Please tick (√) ONE column only.

Question 1:
How confident are you in **providing necessary information** to patients receiving anticoagulation therapy?

| **No.** | **Anticoagulants** | **Not confident at all** | **Not so confident** | **Confident** | **Very confident** |
| --- | --- | --- | --- | --- | --- |
| 1a. | With vitamin k antagonists (VKAs) such as Warfarin |  |  |  |  |
| 1b. | With low molecular weight heparins (LMWHs) |  |  |  |  |
| 1c. | with direct oral anticoagulants (DOACs) such as Dabigatran, Rivaroxaban or Apixaban |  |  |  |  |

Question 2:
How confident do you feel in **discussing the following with patients**?

| **No.** | **Items** | **Not confident at all** | **Not so confident** | **Confident** | **Very confident** |
| --- | --- | --- | --- | --- | --- |
| 2a. | Benefits of anticoagulation therapy |  |  |  |  |
| 2b. | Indications of anticoagulation therapy |  |  |  |  |
| 2c. | Adverse drug reactions of anticoagulation therapy |  |  |  |  |
| 2d. | Management of adverse drug reactions when it comes to anticoagulation therapy (including bleeding risk) |  |  |  |  |
| 2e. | Management of bleeding while on anticoagulation therapy |  |  |  |  |
| 2f. | How to manage missed dose |  |  |  |  |
| 2g. | Monitoring INR and making dosing recommendations based on those values |  |  |  |  |
| 2h. | Management of interactions with anticoagulation therapy |  |  |  |  |
| 2i. | Management of bridging/switching from one anticoagulant to another? |  |  |  |  |

**Section C: Factor(s) affecting confidence level**
What is/are the factor(s) affecting your confidence level in providing pharmaceutical care to patients regarding anticoagulants? (May choose multiple answer)

- Experience in dealing cases needing anticoagulants
- Knowledge about anticoagulants
- Knowledge about the diseases needing anticoagulation therapy
- Previous unfavourable experiences with anticoagulants
- Complexity of anticoagulation therapy
- Others: _______________________________________________________

**Section D: Updates on anticoagulants**
How do you usually obtain the latest information regarding anticoagulants?

- With reference to updated local or international guideline(s)/journals
- With reference to medical website(s) (eg. Medscape Daily News)
- Through attending conferences/workshops
- Through talks organised by pharmaceutical companies
- Continue medical education/Continue pharmacy education by own hospital
- Peer discussion
- With reference to non-medical websites eg Wikipedia
- Others, please specify:__________________________________________

**Section E: Education needed in the area of anticoagulation therapy**

Question 1:
Would you like to receive additional education in the area of anticoagulation therapy?

🞎 Yes; proceed to question 1a 🞎 No; the questionnaire is end

Question 1a:
What areas would interest you in receiving additional education (please select all that apply)?

- Coagulation Pathway
- Medical conditions requiring anticoagulation therapy
- Mechanism of action of anticoagulants
- Interactions (e.g. drug-drug; drug-food) with anticoagulants & their management
- Possible side effects of anticoagulants & their management
- How to assess risk of bleeding
- How to manage bleeding while the patient is on anticoagulants
- Bridging/switching
- Other: Please Specify: _________________________________________

END

**Psychometric properties of original questionnaire**

Validation was done by original authors on section testing on confidence level in providing necessary information on anticoagulants and discussing various aspects in anticoagulation therapy. The psychometric properties of validated section are as below:

 Validity: construct validation was carried out; two components were being identified which are named as basic pharmaceutical care [Question B1a, B1b, B1c, B2a, B2b, B2c] and advanced pharmaceutical care [Question B2d, B2e, B2f, B2g, B2g, B2i]. Rotated factor plotting has clearly shown the individual question being load into the respective components^1^.

 Reliabiltiy: Internal consistency and homogeneity of the section was examined by calculating Cronbach’s alpha. Both components: basic and advanced pharmaceutical care displayed high internal consistency (α = 0.851 and α = 0.877 respectively) in the overall sample from the original study (n = 4212 from 18 countries) with minimal variation at the country level.

References:

1. Papastergiou J, Kheir N, Ladova K, Rydant S. Pharmacists ’ confidence when providing pharmaceutical care on anticoagulants , a multinational survey. Int J Clin Pharm 2017; doi: 10.1007/s11096-017-0551-2.
